# Supplementary material for: Phase glides and self-organization of atomically abrupt interfaces out of stochastic disorder in α-Ga2O3
Source: Nat Commun. 2025 Apr 5;16:3245. doi: 10.1038/s41467-025-58516-9 (PMC11971449; doi:10.1038/s41467-025-58516-9)
Supplement: Supplementary file 1 — Supplementary Information [file 41467_2025_58516_MOESM1_ESM.pdf]

Supplementary Information

Phase glides and self-organization of atomically abrupt interfaces out of stochastic disorder in α-Ga_2O_3

Alexander Azarov[1*], Javier García Fernández[1], Junlei Zhao[2^], Ru He[3], Ji-Hyeon Park[4], Dae-Woo Jeon[4], Øystein Prytz[1], Flyura Djurabekova[3], and Andrej Kuznetsov[1#]

[1] University of Oslo, Department of Physics, Centre for Materials Science and Nanotechnology, PO Box 1048 Blindern, N-0316 Oslo, Norway

[2] Department of Electrical and Electronic Engineering, Southern University of Science and Technology, Shenzhen 518055, China

[3] Department of Physics and Helsinki Institute of Physics, University of Helsinki, P.O. Box 43, FI-00014, Finland

[4] Korea Institute of Ceramic Engineering & Technology, Jinju 52851, South Korea

Table of Contents

Supplementary note 1: E_p-V curves of the α, γ, and amorphous Ga_2O_3 phases 1

Supplementary note 2: PRDF of α-Ga_2O_3 and γ-Ga_2O_3 phases 2

Supplementary note 3: α-to-γ Ga_2O_3 phase transition induced by Au ions 3

Supplementary note 4: Separation of phase transition and surface amorphization 4

[*] alexander.azarov@smn.uio.no
[^] zhaojl@sustech.edu.cn
[#] andrej.kuznetsov@fys.uio.no

Supplementary note 1: E_p-V curves of the α, γ, and amorphous Ga_2O_3 phases

As shown in Supplementary Figure 1, the potential energy-volume (E_p-V) curves of the α, γ, and amorphous Ga_2O_3 phases at zero temperature and hydrostatic strain are calculated using the ML-tabGAP potential [1]. The amorphous cells are prepared at different densities using a conventional melt-quenching scheme [2]. For each density, five independent stoichiometric cells containing 1,000 randomly generated atoms are

first heated up to 3,000 K and equilibrated for 50 ps. Subsequently, the structures are cooled down to 0.1 K at a constant cooling rate of 10 K/ps and eventually optimized to a local minimum (stopped at an atomic-force tolerance of 10^{-5} eV/Å).

The dashed line in Supplementary Figure 1 represents the common tangent to the E_p-V curves for both the α and γ phases, indicating the purely thermodynamically driven α-to-γ phase transition path of state. The shaded area in Supplementary Figure 1 denotes the overall amorphous region, considering the potential plastic deformation of the amorphous phase under strain.

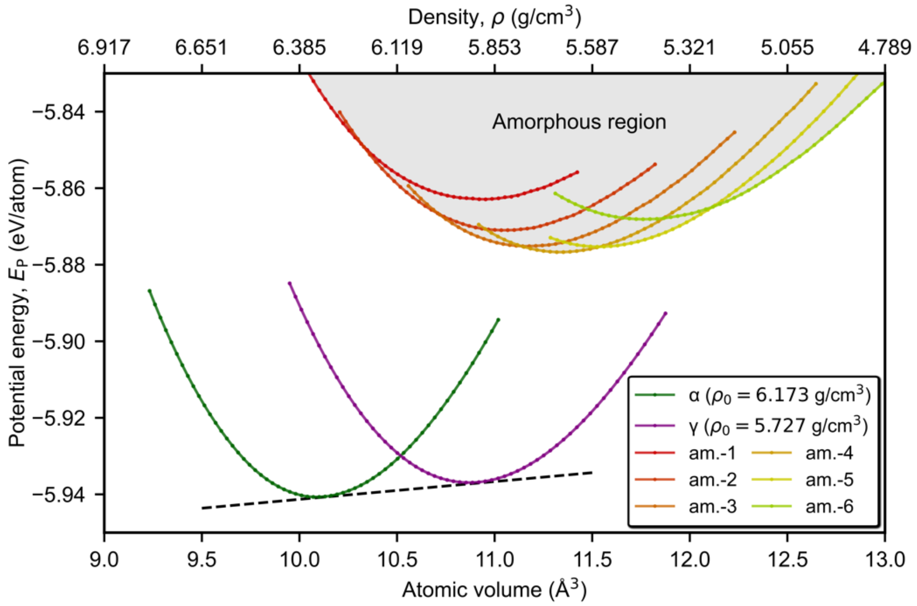

Supplementary Figure 1. Potential energies as a function of atomic volumes/densities are calculated using the ML-tabGAP potential [1]. The dashed line represents the common tangent to the E_p-V curves for both the α and γ phases. Notably, the overall amorphous region (shaded area) is approximately delineated by an envelope curve of all the E_p-V curves corresponding to the amorphous Ga_2O_3 cells optimized at different densities. Five independent amorphous cells are simulated for each density to obtain the average value of E_p.

Supplementary note 2: PRDF of α-Ga_2O_3 and γ-Ga_2O_3 phases

Supplementary Figure 2(a) presents snapshots of the close-packed oxygen planes in α-Ga_2O_3 and γ-Ga_2O_3, each containing the same number of atoms. The width and height of these planes show no significant differences compared to the stacking perpendicularly to the close-packed direction depicted in Fig. 4(a). In Supplementary Figure 2(b), the partial radial pair distribution function (PRDF) curves of the close-packed oxygen sublattice planes in α and γ-Ga_2O_3 are displayed, with overlapping peaks, indicating that the dimensions of the close-packed oxygen planes in both phases are nearly identical. However, distinct differences are evident in the shapes of the peaks in the PRDF curves, particularly at shorter distances. The sharper peaks

observed in α-Ga_2O_3 are due to its well-defined crystal structure, whereas γ-Ga_2O_3 exhibits a more defective structure. The PRDF curve of γ-Ga_2O_3 exhibits a single peak for the nearest neighbor distances, while α-Ga_2O_3 shows two peaks. As illustrated in the pristine α-Ga_2O_3 snapshots (Fig. 4(d)), one of the three octahedral interstices in the hcp O sublattice is vacant, causing the nearby O atoms to be closer to each other than those surrounding the Ga atoms, resulting in two distinct peaks in the PRDF curves. Although the peak shapes of the PRDF curves differ between the two phases due to their differing crystal structures, the peak positions are similar, especially at longer distances. This indicates no significant "isotropic" expansion within the close-packed oxygen sublattice planes between γ and α-Ga_2O_3.

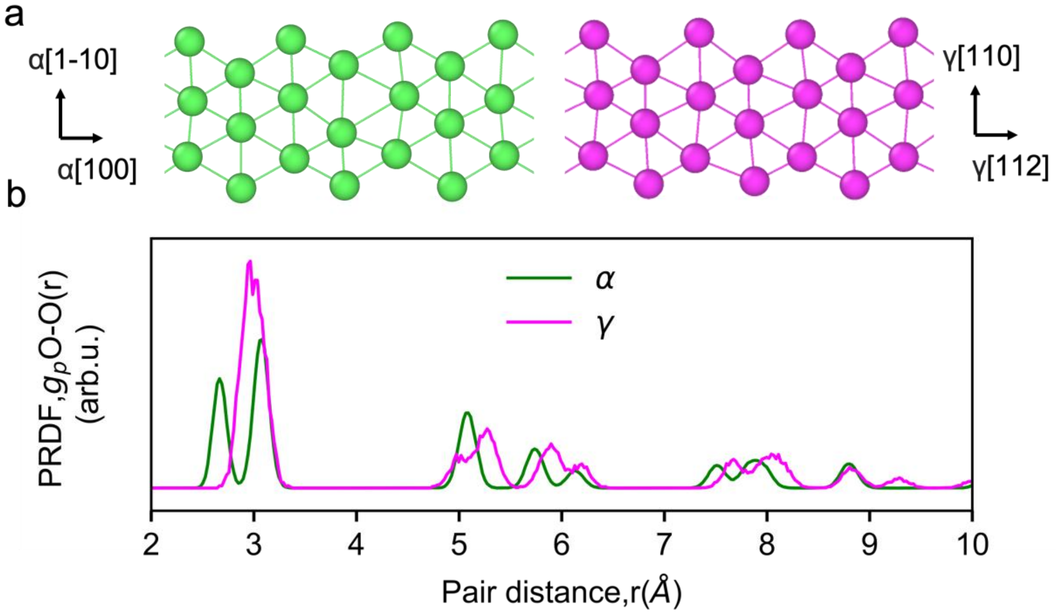

Supplementary Figure 2 (a) Close-packed planes of *hcp* oxygen sublattice in α-Ga_2O_3 (left) and *fcc* oxygen sublattice in γ-Ga_2O_3 (right). (b) The PRDF curves of the close-packed planes of the oxygen sublattice in α-Ga_2O_3 (red) and γ-Ga_2O_3 (green).

Supplementary note 3: α-to-γ Ga_2O_3 phase transition induced by Au ions

In order to exclude options of possible chemical or doping effects on the surface amorphization and ion-induced phase transformation in α-Ga_2O_3, we performed additional Au ion implants, since Au supposed to be chemically inert in this matrix and as such providing a comparative reference point. For that matter, Supplementary Figure 3 shows (a) Rutherford backscattering spectrometry in channeling mode (RBS/C) and (b) corresponding x-ray diffraction (XRD) 2θ scans of the α-Ga_2O_3 samples implanted with 1.2 MeV $^{197}Au^{+}$ ions to a dose of 1×10^{16} cm^{-2}, corresponding to the disorder of

~130 dpa. As seen from the data, this disordering was sufficient to form the γ-Ga_2O_3 layer. Indeed, XRD 2θ scan of the Au implanted sample (see panel (b)) includes a (222) γ-Ga_2O_3 reflection similar to that in Fig. 2(b) in the main part of the manuscript. Moreover, RBS data in panel (a) demonstrate very similar features as those for the high dose Ni implanted samples, see Fig.2(a) in the main part of the manuscript. Specifically, enhanced RBS yield near the surface is assigned to the amorphous layer, followed-up by an inclined box shaped profile corresponding to the γ-layer. Thus, this similarity of the data obtained with Au contra Ni implants indicate that both the surface amorphization and α-to-γ phase transition in α-Ga_2O_3 are the disorder-induced phenomena with minor, if any, impacts of chemical effects. Notably, the enhanced yield near the surface in the random and channeling RBS spectra of the Au-implanted sample is because of the backscattering from Au, while the γ-layer thickness variation for the γ-layers obtained using the same dpa with Au and Ni implants correlates with higher collision density for heavier ions [3].

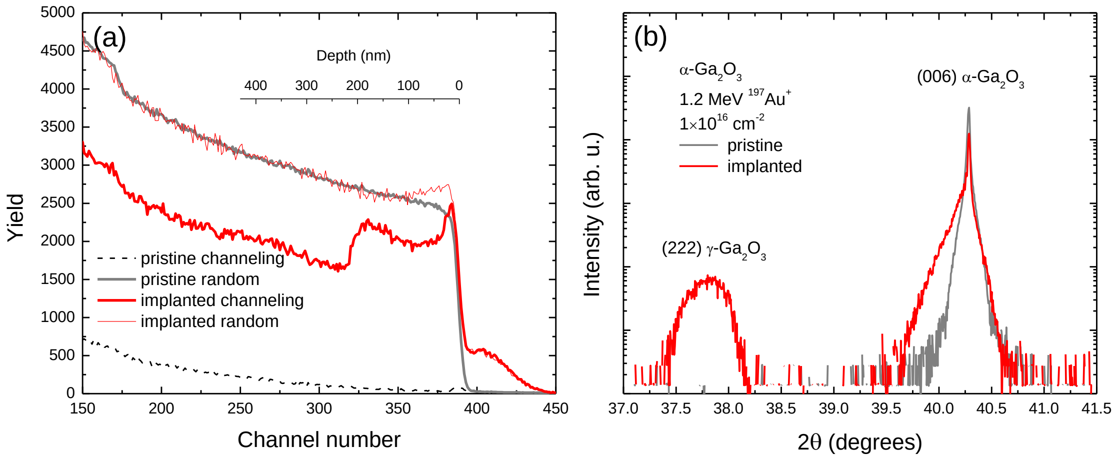

Supplementary Figure 3 (a) RBS/C spectra and (b) corresponding XRD 2θ scans of the α-Ga_2O_3 samples irradiated with 1.2 MeV Au ions up to a dose of 1×10^{16} cm^{-2}. The RBS/C spectrum for the pristine sample is shown by a dashed line in panel (a) for comparison.

Supplementary note 4: Separation of phase transition and surface amorphization

The data in Fig. 2 of the main text show that the surface amorphous layer and the region where the γ-phase form rather close to each other. Thus, in order to investigate whether there is an interplay between the surface amorphization and the phase transformation, we performed additional 800 keV Ni implants, so that the primary defect generation region is located twice deeper in the sample as compared to that in Fig. 2 of the main text. Thus, for comparison, Supplementary figure 4 shows (a) RBS/C and (b) XRD data for this higher energy implants. As seen from panel (a), 800 keV Ni

implant results in the disorder profile with two characteristic regions located near the surface and close to the R_{pd} corresponding to 280 nm according to the SRIM code [4] simulations. In accordance with the data in Fig. 2, similarly here, the near surface RBS/C peak may be assigned to a thin amorphous layer. In its turn, the RBS/C peak at 300-350 nm in panel (a) can be attributed to a γ-layer, in correlation with the XRD observation of the (222) γ-Ga_2O_3 reflection in panel (b). Importantly, here, in contrast to the data in Fig.2 in the main text, the γ-layer and the amorphous region are well separated, and for sure not affecting each other by their proximity. Thus, from this comparison, we conclude that there is no compulsory interplay of the amorphization with the polymorph transformation or vise versa.

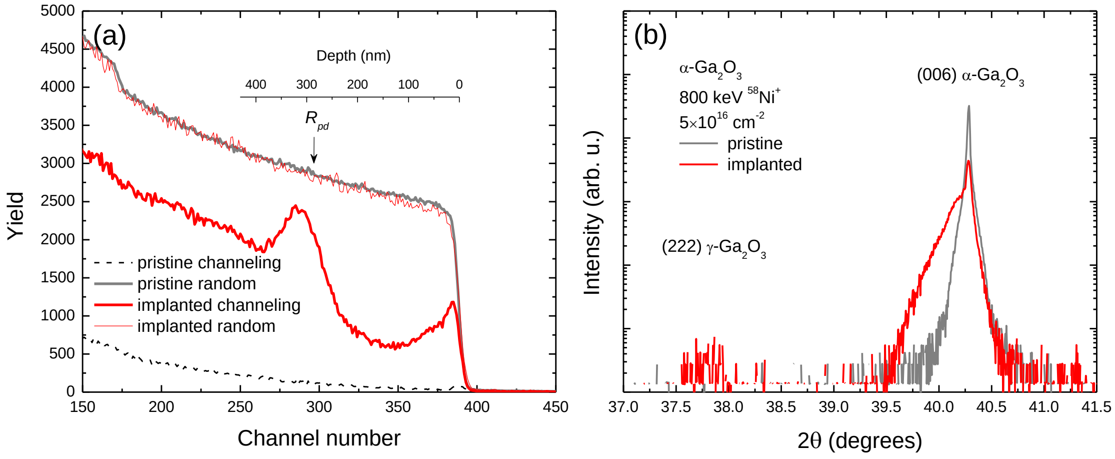

Supplementary Figure 4 (a) RBS/C spectra and (b) corresponding XRD 2θ scans of the α-Ga_2O_3 samples irradiated with 800 keV Ni ions up to a dose of 5×10^{16} cm^{-2}. The channeling pristine spectrum is shown by a dashed line in panel (a) for comparison.

Supplementary References:

1. Zhao, J.; Byggmästar, J.; He, H.; Nordlund, K.; Djurabekova, F.; and Hua, M. "Complex Ga_2O_3 polymorphs explored by accurate and general-purpose machine-learning interatomic potentials", *npj Comput. Mater.* **9** (2023) 159.
2. Zhang, J.; Zhao, J.; Byggmästar, J.; Frankberg E. J.; and Kuronen, A. "Large-scale atomistic study of plasticity in amorphous gallium oxide with a machine-learning potential", *arXiv* (2024), DOI: 10.48550/arXiv.2404.17353.
3. Klevtsov, A.I.; Karaseov, P.; Azarov, A.; Karabeshkin, K.; Fedorenko, E.; Titov, A.; and Kuznetsov, A. "Nonlinear effects in α-Ga_2O_3 radiation phenomena", *APL Materials* **12** (2024) 111121.
4. J. F. Ziegler, M. D. Ziegler, and J. P. Biersack, "SRIM—the stopping and range of ions in matter (2010)", *Nucl. Instrum. Methods Phys. Res., Sect. B* **268** (2010) 1818.
