# Supplementary material for: Phase glides and self-organization of atomically abrupt interfaces out of stochastic disorder in α-Ga2O3
Source: Nat Commun. 2025 Apr 5;16:3245. doi: 10.1038/s41467-025-58516-9 (PMC11971449; doi:10.1038/s41467-025-58516-9)
Supplement: Supplementary file 2 — Description of Additional Supplementary Files [file 41467_2025_58516_MOESM2_ESM.pdf]

Description of Additional Supplementary Files

File Name: Supplementary Movie 1

Description: Supplementary Movie 1 includes molecular dynamics simulations of the interface transition towards atomically sharp γ/α interface
